# Targeting Modifiable Risks: Molecular Mechanisms and Population Burden of Lifestyle Factors on Male Genitourinary Health

**DOI:** 10.3390/ijms26199698

**Published:** 2025-10-05

**Authors:** Xingcheng Yang, Meiping Lan, Jiawen Yang, Yuyi Xia, Linxiang Han, Ling Zhang, Yu Fang

**Affiliations:** 1 Department of Clinical Medicine, School of Medical, Wuhan University of Science and Technology, Wuhan 430065, China; yangxingcheng@wust.edu.cn (X.Y.); hanlinxiang@wust.edu.cn (L.H.); 2 Department of Environmental Hygiene and Occupational Medicine, School of Public Health, Wuhan University of Science and Technology, Wuhan 430065, China; 19585424574@163.com (M.L.); yangjiawen@wust.edu.cn (J.Y.); xiayuyi@wust.edu.cn (Y.X.)

**Keywords:** health risk factor, suboptimal lifestyle, male health, urogenital system

## Abstract

Health represents a state of complete physical, mental, and social well-being, with lifestyle factors accounting for approximately 60% of health determinants. Suboptimal health describes an intermediate condition between wellness and disease. According to 2023 WHO data, infertility affects approximately 17.5% of global adults, with male factors implicated in 30–50% of cases, establishing infertility as a critical public health challenge. Substantial preclinical and clinical evidence links suboptimal lifestyles to male reproductive dysfunction, positioning these behaviors as modifiable infertility risk factors encompassing environmental contaminants and lifestyle patterns. This systematic review synthesizes evidence on five key lifestyle determinants—tobacco, alcohol, microplastics, sedentariness, and sleep disruption—affecting male genitourinary health. Adopting an evidence-based medicine framework, we integrate epidemiological and experimental research to establish foundational knowledge for developing novel preventive strategies targeting male suboptimal health.

## 1. Introduction

Male reproductive health is a critical component of overall well-being, with profound implications for individual quality of life, family planning, and broader societal health [1]. It encompasses not only physiological function but also psychological and social dimensions, making it a central issue in men’s health. The genitourinary system encompasses organs such as the kidneys, bladder, urethra, prostate, testes, and penis, each playing critical roles in filtration, excretion, reproduction, and hormonal regulation. Key measurable outcomes reflecting their function include renal function markers (e.g., glomerular filtration rate, creatinine clearance), semen parameters (sperm count, motility, morphology), hormonal profiles (testosterone, luteinizing hormone, prostate-specific antigen), and urinary metrics (volume, proteinuria, voiding frequency). However, global male reproductive health indicators are concerning: WHO data indicates approximately 15% of reproductive-aged couples experience infertility, with male factors contributing 20–70% [2]. Over the past 50 years, significant declines in human sperm quality have occurred, with parameters decreasing over 50%, alongside rising clinical incidence of oligozoospermia, asthenozoospermia, and teratozoospermia [3]. Concurrently, the burden of urological diseases is increasing; urological malignancies (e.g., prostate, bladder cancer) rank highly in global male cancer morbidity and mortality, presenting risks across the lifespan [4]. Crucially, paternally inherited abnormalities can impact offspring health via epigenetic mechanisms [5]. Addressing the men’s health crisis demands systemic strategies, from urgent medical intervention to ensuring long-term socio-demographic sustainability, making it a priority research and policy area.

Substantial evidence indicates that lifestyle factors constitute a major, potentially modifiable determinant of men’s health outcomes, often outweighing the contribution of healthcare access itself [6]. Classified by ICD-11 and public health bodies, a ‘suboptimal lifestyle’ denotes a deviation from equilibrium, encompassing nutritional imbalance (e.g., high-fat, low-carbohydrate diets), physical inactivity (>8 h sedentary/day), sleep deficiency (<6 h/day), chronic psychological stress, and addictive behaviors (tobacco/alcohol dependence) [7]. Research confirms that traditional and novel environmental pollutants synergize with modern lifestyles to impair male genitourinary function [8]. Crucially, unlike relatively immutable genetic factors, the modifiable nature of suboptimal lifestyles presents a pivotal intervention target for addressing the men’s health crisis. Consequently, implementing primary prevention through lifestyle medicine is urgently needed to catalyze a paradigm shift from reactive treatment towards proactive health promotion.

## 2. Cigarette Smoking: Dose-Dependent Urogenital Toxicity and Spermatogenic Impairment

**Figure 1:**
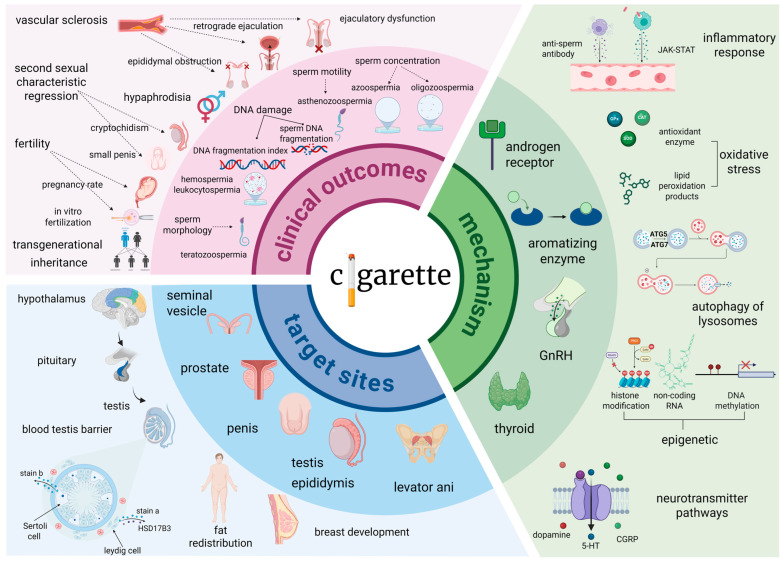
Male smoking and fertility risk: pathophysiological mechanisms and clinical evidence. Clinical outcomes manifesting as reduced sperm quality, sexual dysfunction, impaired fertility, and intergenerational risks. Affected sites encompass the testes, epididymis, prostate, and endocrine system. Key mechanistic pathways involve hormonal disruption, inflammation, oxidative stress, dysregulated autophagy, and epigenetic modifications. GnRH, Gonadotropin-Releasing Hormone; CGRP, Calcitonin Gene-Related Peptide; JAK-STAT, Janus Kinase-Signal Transducer and Activator of Transcription; ATG5, Autophagy-related gene 5; ATG7, Autophagy-related gene 7; HSD17B3, Hydroxysteroid 17-beta dehydrogenase 3; 5-HT, 5-Hydroxytryptamine; SAM, S-Adenosyl methionine; PRC2, Polycomb Repressive Complex 2; RNAPⅡ, RNA Polymerase II; Me3, Trimethylation.

Tobacco smoke delivers a complex mixture of toxicants (e.g., nicotine, Cd, Pb) with established gonadotoxic and carcinogenic effects, exhibiting marked male predominance in global prevalence (25% males vs. 5.4% females) [9], particularly in China (26.7–27.2% male smokers) [10]. Clinically, smoking demonstrates a dose/time-dependent association with urological pathology (Figure 1). Current smokers face a 17–22% increased risk of kidney stones and a 9–34% elevated risk of chronic kidney disease (CKD), with prenatal/childhood exposure conferring the highest CKD risk (HR = 1.34, *p* < 0.001) [11]. Male smokers exhibit accelerated renal decline, including a 24% higher CKD progression risk, 5.5–6.9% reduced eGFR and 1.6–2.55-fold increased proteinuria risk (*p* < 0.05) [12,13,14]. For urological malignancies, smoking exhibits a biphasic effect—prostate cancer incidence paradoxically decreases (RR = 0.74, *p* < 0.001) [15] but mortality surges 42% (RR = 1.42, *p* < 0.001) [16], with recent quitters (<15 years) facing a 233% risk escalation (OR = 3.33, *p* < 0.01) [17]. Conversely, bladder cancer risk increases nonlinearly, plateauing at 3.27-fold beyond 20 cigarettes/day and reaching 15.8-fold with ≥20 pack-years (AOR = 15.8, *p* < 0.001) [18,19,20].

Beyond renal pathologies, smoking exerts pronounced dose-dependent damage on male reproductive function, specifically targeting spermatogenesis (Figure 1). Smokers demonstrate significant reductions in sperm concentration, motility, and morphology (reported ORs ranging from 0.84 to 0.89), with heavy smokers (>10 cigarettes/day) showing 13.7% lower concentration and 7.1% reduced progressive motility [21,22,23,24]. Notably, smokeless tobacco users exhibit a 24% decrease in total sperm count despite a 14% testosterone elevation [25], implicating direct gonadal toxicity independent of endocrine disruption. Critically, this reproductive impairment demonstrates reversibility: 3 months of abstinence increases semen volume (16.9%), sperm concentration (22.7%), and total sperm count (44.5%) [26].

These divergent effects reflect tobacco’s dual-phase mechanistic profile. Acute nicotine exposure transiently inhibits tumor growth via neuroendocrine modulation (e.g., dopamine pathways), explaining early paradoxical cancer suppression [27]. Conversely, chronic accumulation of nitrosamines and polycyclic aromatic hydrocarbons drives sustained organ dysfunction, carcinogenesis, and spermatogenic impairment through oxidative stress, DNA damage, and inflammation (Figure 1) [28]. However, antioxidants such as protocatechuic acid (PCA) can mitigate lipid peroxidation and DNA damage by upregulating the expression of antioxidant enzymes including heme oxygenase-1 (HO-1), superoxide dismutase 2 (SOD2), and nitrosylquinoline oxidase 1 (NQO1). Furthermore, by activating the AMPK/PGC-1α/Nrf1 pathway, they improve mitochondrial function, thereby restoring sperm motility and testosterone levels [29]. This biphasic toxicity model unifies the observed clinical spectrum from dose-dependent renal decline and cancer paradoxes to reversible spermatogenic deficits underscoring cessation as a critical intervention point.

Collective evidence indicates that tobacco exposure impairs male reproductive function through multiple pathways, with the severity of impairment exhibiting a dose-dependent relationship. Notably, smoking cessation has been demonstrated to effectively ameliorate sperm parameters.

## 3. Ethanol Exposure: Organ-Specific Pathophysiology and Genetic Polymorphism-Mediated Effects

**Figure 2:**
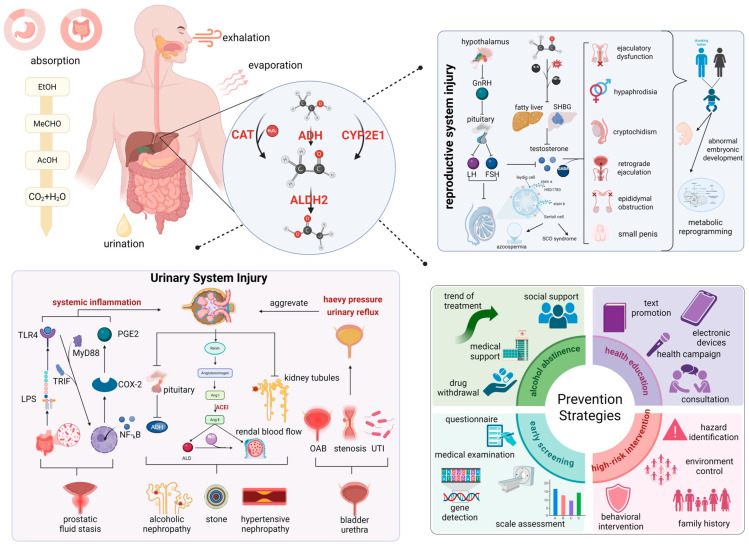
Alcohol exposure: multi-organ pathological damage in men. Urological sequelae encompass renal ischemic damage, electrolyte imbalance, bladder detrusor dysregulation causing overactivity, heightened urethral infection/stricture risk, and prostatic reactions. Reproductive impacts include sexual dysfunction, azoospermia, diminished testosterone, and offspring developmental disorders with metabolic reprogramming via epigenetic mechanisms. Preventive strategies include controlled intake, hydration, biomarker surveillance, and early high-risk group intervention. CAT, Catalase; ADH: Antidiuretic Hormone; SHBG, Sex Hormone-Binding Globulin; GABA, Gamma-Aminobutyric Acid; SCO, Sertoli Cell-Only Syndrome; OAB, Overactive Bladder; UTI, Urinary Tract Infection; ACEI, Angiotensin-Converting Enzyme Inhibitor; LPS, Lipopolysaccharide; CYP2E1, Cytochrome P450 2E1; ALDH2, Aldehyde dehydrogenase 2; ALD, Aldehyde dehydrogenase; ADH, Alcohol dehydrogenase; TLR4, Toll-like receptor 4; TRIF, TIR-domain-containing adapter-inducing interferon-β; MyD88, Myeloid differentiation primary response 88; PGE2, Prostaglandin E2; COX-2, Cyclooxygenase-2; NF-kB, Nuclear factor kappa-light-chain-enhancer of activated B cells.

In 2015, global epidemiological data revealed an average per capita alcohol intake of 6.42 L of pure ethanol equivalents among adult populations [30]. Cross-sectional surveys indicated that 18.3% of adults exhibited binge drinking behavior (operationally defined as single-occasion consumption exceeding 60 g of absolute ethanol) during the preceding 30-day monitoring period [31]. Ethanol is rapidly absorbed in the gastrointestinal tract and distributed systemically, with hepatic metabolism primarily mediated by alcohol dehydrogenase (ADH) and catalase (CAT), oxidizing ethanol to acetaldehyde. This genotoxic intermediate is detoxified by aldehyde dehydrogenase 2 (ALDH2) to acetate, while cytochrome P450 2E1 (CYP2E1) generates reactive oxygen species during microsomal oxidation, collectively establishing pro-inflammatory and oxidative milieus that drive urogenital toxicity prior to renal excretion (Figure 2).

The impact of ethanol exposure on urogenital disorders exhibits significant organotropism and population-stratified susceptibility, mediated through distinct pathophysiological pathways across anatomical compartments (Figure 2). Epidemiologic stratification of renal disease demonstrated a 23% risk reduction for CKD in males with moderate ethanol intake (70 g/d; RR = 0.78) [32]. However, this nephroprotective association underwent complete age-dependent attenuation beyond the sixth decade (RR = 1.00) [32], suggesting metabolic senescence alters ethanol’s pharmacodynamic profile. Paradoxical findings emerged in nephrolithiasis risk assessments. Cross-sectional surveys identified inverse correlations with heavy ethanol consumption (OR = 0.76), whereas Mendelian randomization studies revealed positive associations between drinking frequency (OR = 1.29) and urolithiasis incidence, potentially indicating cumulative metabolic perturbations override ethanol’s transient diuretic effects [33]. Bladder pathophysiology demonstrates critical population stratification patterns. Moderate (RR = 0.97, *p* = 0.59) or heavy (RR = 1.07, *p* = 0.58) alcohol consumption was not associated with the disease in Western populations, yet elevated susceptibility in Japanese populations (RR = 1.31, *p* < 0.01) and spirit-preferring males (RR = 1.42–1.50) [34]. Conversely, drinking ≥10 alcoholic drinks per month was associated with a 59% lower risk of detrusor overactivity (OR = 0.41) [35], which may be mediated by concentration-dependent modulation of bladder smooth muscle γ-aminobutyric acid receptors by ethanol. Prostatic disease analyses demonstrated absence of dose–response relationships between cumulative ethanol–exposure and adenocarcinoma risk (RR = 1.00) [36]. However, developmental exposure assessments identified 3.21-fold elevated odds (OR = 3.21) for high-grade prostate cancer with adolescent ethanol consumption ≥7 sessions/week [37], suggesting androgen-sensitive epithelial plasticity during pubescence. Biochemical surveillance studies documented ethanol-induced suppression of free prostate-specific antigen (fPSA) levels (β = −0.11, *p* < 0.05) and significant ethanol dietary inflammation interaction (*p* = 0.037) [38,39], raising clinical concerns about compromised diagnostic sensitivity in ethanol-consuming populations undergoing PSA-based malignancy screening.

Ethanol exposure manifests multiphasic dose–response relationships with male reproductive pathophysiology, characterized by testicular parenchymal remodeling and spermatozoal quality deterioration (Figure 2). Bilateral testicular atrophy in chronic ethanol consumers, with moderate (Δ = −4.164 mL, *p* < 0.001) and heavy intake cohorts (Δ = −5.500 mL, *p* < 0.001) demonstrating progressive volumetric loss [40]. Frequency-dependent effects were evident with subjects consuming ethanol ≥12 times/week exhibiting 3.451 mL reduced testicular volume versus ≤11 times/month counterparts (*p* < 0.001), suggesting hypothalamic–pituitary–gonadal axis disruption [40]. Meta-analysis of 23,258 hormonal profiles confirmed ethanol-induced endocrine perturbation: suppressed testosterone (SMD = −1.60), follicle-stimulating hormone (FSH; SMD = −0.47), and luteinizing hormone (LH; SMD = −1.35), coupled with elevated estradiol (SMD = 0.22), collectively establishing an anti-spermatogenic steroid milieu [41]. These neuroendocrine alterations directly mediate spermatogenic impairment, evidenced by ethanol-associated declines in seminal volume (Δ = −0.25 mL, 95% CI 0.07–0.42) and morphologically normal spermatozoa (Δ = −1.87%, 95% CI 0.86–2.88). Daily consumers exhibited 5.17% elevated teratozoospermia risk (*p* = 0.03) [42], corroborated by multicenter studies showing concentration-dependent oligozoospermia (OR = 3.72/2.32) [43,44]. Polymorphism-dependent vulnerability was observed in kinetic parameters: ALDH2 allele carriers exhibited precipitous motility decline (43%→20%, *p* = 0.005) post exposure [45], while Han Chinese cohorts demonstrated median total motility reduction (64%→56%, *p* = 0.001) [46]. Nonlinear dose–response patterns emerged in controlled intake scenarios. Dual independent investigations identified J-curve associations with moderate ethanol consumption (4–7 units/week), showing enhanced seminal volume (3.0 vs. 2.4 mL), concentration (31 vs. 24.5 × 10^6^/mL), and total sperm count (87.9 vs. 51.5 × 10^6^/mL) [42,47]. Mechanistic studies implicate oxidative–genotoxic pathways, with murine models exhibiting 52% increased spermatozoal cephalic anomalies (10.5% vs. 6.9%, *p* < 0.01) and accelerated nuclear chromatin decondensation (57.1% vs. 48.3%, *p* < 0.05) [48]. Clinical cohorts demonstrated ethanol dose-dependent sperm DNA fragmentation index elevation (Δ = +5.83%, *p* = 0.002) and corresponding live birth rate reduction (OR = 0.5) [49]. Contradictory findings regarding DNA integrity suggest potential non-oxidative toxicity mechanisms, possibly involving histone modification or spermatozoal miRNA dysregulation [50,51]. Concurrently, alcohol or high-fat diets further induce systemic inflammation and endotoxinaemia by precipitating obesity and gut microbiota dysbiosis. However, the core mechanism ultimately leading to male reproductive dysfunction is oxidative stress damage within the testes, rather than endotoxins themselves [52]. Alcoholic liver injury triggers a systemic inflammatory response through a ‘multiple-hit’ mechanism, allowing pro-inflammatory cytokines (such as TNF-α and IL-6) to infiltrate the testes. This disrupts the blood–testis barrier and suppresses Leydig cell function. Excessive reactive oxygen species (ROS) produced by the liver also induce systemic oxidative stress, collectively causing remote damage to the testes’ hormone synthesis and spermatogenesis environment [53].

Ethanol exerts multifactorial effects on male genitourinary systems, necessitating clinical frameworks that integrate comprehensive assessments of genetic polymorphisms and metabolizing enzyme activities when evaluating toxicological mechanisms and formulating targeted interventions (Figure 2).

## 4. Microplastics and Male Health: Multifaceted Mechanisms, Particle Size-Dependent Effects, and Transgenerational Implications

**Figure 3:**
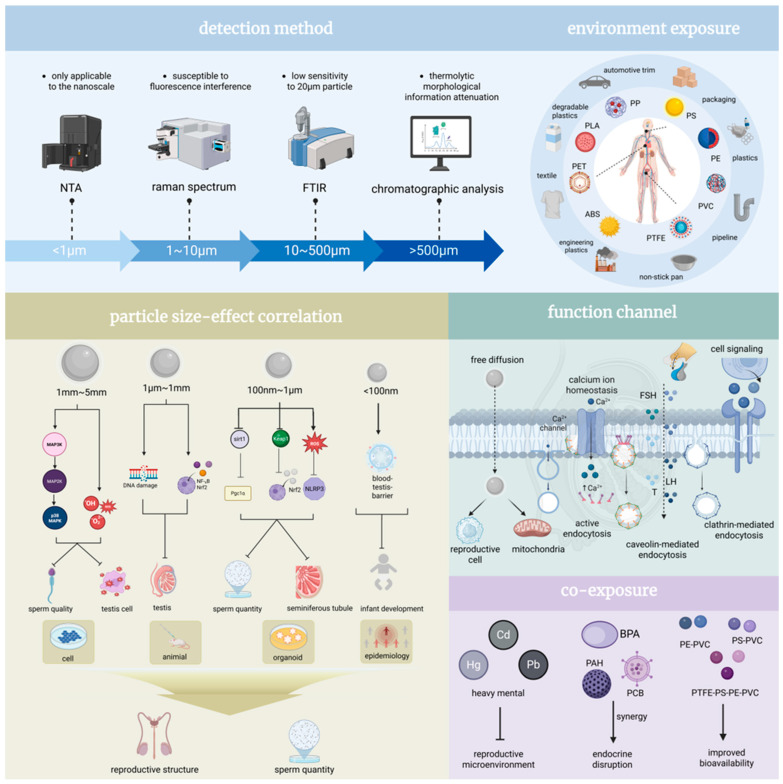
Size-dependent impacts of microplastics on male urogenital health. Human exposure pathways associated with diverse microplastic types; detection methodologies applicable across varying particle size fractions, coupled with intrinsic strengths and limitations; size-dependent associations between microplastic properties and male health outcomes, alongside the mechanisms; and synergistic interactions resulting from concurrent exposure to microplastics and co-contaminants. NTA, Nanoparticle Tracking Analysis; FTIR, Fourier Transform infrared spectroscopy; Pgc1α, Peroxisome proliferator-activated receptor γ coactivator 1-α; Nrf2, Nuclear Factor erythroid 2-Related Factor 2; NLRP3, NOD-like receptor thermal protein domain associated protein 3; PP, Polypropylene; PS, Polystyrene; PE, Polyethylene; PVC, Polyvinyl chloride; PTFE, Polytetrafluoroethylene; ABS, Acrylonitrile butadiene styrene; PET, Polyethylene terephthalate; PLA, Polylactic acid; PP, Protein phosphatase; MAP2K, Mitogen-activated protein kinase kinase; MAP3K, Mitogen-activated protein kinase kinase kinase; P38, p38 Mitogen-activated protein kinase; MARK, MAP/microtubule affinity-regulating kinase; NF-fB, Nuclear factor kappa-light-chain-enhancer of activated B cells; sir1, Silent information regulator 1; keap1, Kelch-like ECH-associated protein 1; ROS, Reactive oxygen species; T, Testosterone; BPA, Bisphenol A; PAH, Polycyclic aromatic hydrocarbo; PCB, Polychlorinated biphenyl; PE-PVC, Polyethylene-Polyvinyl chloride; PS-PVC, Polystyrene-Polyvinyl chloride; PTFE-PE-PVC, Polytetrafluoroethylene-Polyethylene-Polyvinyl chloride.

Microplastics (MPs), defined as plastic particles <5 mm in diameter, are ubiquitously distributed across marine ecosystems, terrestrial soils, atmospheric aerosols, and biological matrices (Figure 3). They predominantly comprise polymers including polyethylene (PE), polystyrene (PS), polyvinyl chloride (PVC), and polypropylene (PP) [54]. Common sources encompass degradation of plastic waste, industrial effluent discharge, and microbeads from personal care formulations. Due to their minute dimensions, high specific surface area, and hydrophobic nature, MPs exhibit a pronounced propensity to adsorb environmental endocrine-disrupting chemicals (EDCs), heavy metals, and pathogenic microorganisms, thereby forming complex composite contaminants [55,56,57,58]. Studies confirm MPs enter organisms via ingestion, inhalation, or dermal absorption, accumulate within organs, and disrupt physiological homeostasis, with reproductive system toxicity being particularly salient.

The mechanistic complexity and key exposure determinants critically govern the reproductive toxicity profile of microplastics. MP-induced reproductive toxicity involves multifaceted pathways, notably oxidative stress, inflammatory cascades, endocrine disruption, and apoptotic signaling. Toxicity exhibits distinct size-dependent variations: microplastics within the size range of 1–5 mm significantly impair sperm motility, viability, and serum testosterone levels by activating the P38 MAPK signaling pathway and inducing oxidative stress, culminating in testicular histopathology [59,60,61]. Microplastics ranging from 1 μm to 5 mm provoke mitochondrial dysfunction, compromise the blood–testis barrier integrity, and induce DNA damage primarily via the NF-κB/Nrf2/HO-1 pathway, leading to diminished sperm count and quality. Microplastics within the size range of 100 nm–1 μm directly inflict structural damage to seminiferous tubules and reduce spermatogenic cell populations [62]. Microplastics smaller than 100 nm, owing to their reduced size, demonstrate enhanced capacity for cellular membrane penetration and testicular accumulation, resulting in more severe reproductive impairment (Figure 3) [63,64,65]. Dose and exposure duration are pivotal determinants; chronic low-dose exposure (e.g., 100 µg/L over 180 days) elicits progressive sperm quality deterioration [66], whereas acute high-dose exposure (e.g., 2000 mg/kg for 28 days) rapidly triggers inflammation and hormonal dysregulation [67]. Dose–response relationships are evident, exemplified by PS-MPs where minimal effects occur at 20 µg/L, escalating to maximal toxicity at 2000 µg/L [68,69,70]. Additives like di(2-ethylhexyl) phthalate (DEHP) and bisphenol A (BPA) potentiate toxicity: DEHP disrupts testicular immune microenvironment homeostasis, promotes Th2/Th17 immune polarization, and inhibits meiotic progression via the Trp53/p38-MAPK pathway [71,72,73,74,75]. BPA impairs germ cell proliferation and reduces sperm quality through activation of the TLR4/NF-κB signaling cascade [76,77,78,79,80]. Furthermore, MP co-exposure with heavy metals (e.g., cadmium) [81], microcystins (MCLR), or arsenic generates synergistic effects [82], amplifying oxidative stress and DNA damage, ultimately manifesting as testicular lesions and increased sperm malformation rates (Figure 3) [83,84,85,86,87].

Experimental models robustly confirm the reproductive toxicity of microplastics. Rodent studies demonstrate MP exposure reduces serum testosterone and gonadotropin (LH, FSH) levels, depletes spermatogenic cells, and compromises blood–testis barrier function [88,89]. Aquatic models (e.g., zebrafish, oysters) exhibit gonadal atrophy, diminished sperm motility, and reduced offspring viability [90,91,92]. Recent animal studies have revealed that 80 nm nanoplastics impair spermatogonial proliferation by inhibiting Cyp26a1, a key gene in retinoic acid metabolism, whilst 5 μm microplastics disrupt energy metabolism by downregulating the thyroid hormone receptor Thra [93]. Clinical manifestations in exposed models include sperm head malformations, reduced motility, testicular fibrosis, and infertility [65,94,95]. While mechanistic insights are emerging, human epidemiological data remain scarce. Future research imperatives include elucidating MP metabolic fate, establishing definitive dose thresholds, and characterizing long-term transgenerational consequences to inform effective protective strategies for vulnerable populations.

## 5. Health Risks Associated with Sleep Disorders in Men: Epidemiological Evidence, Disruption Mechanisms and Clinical Implications

**Figure 4:**
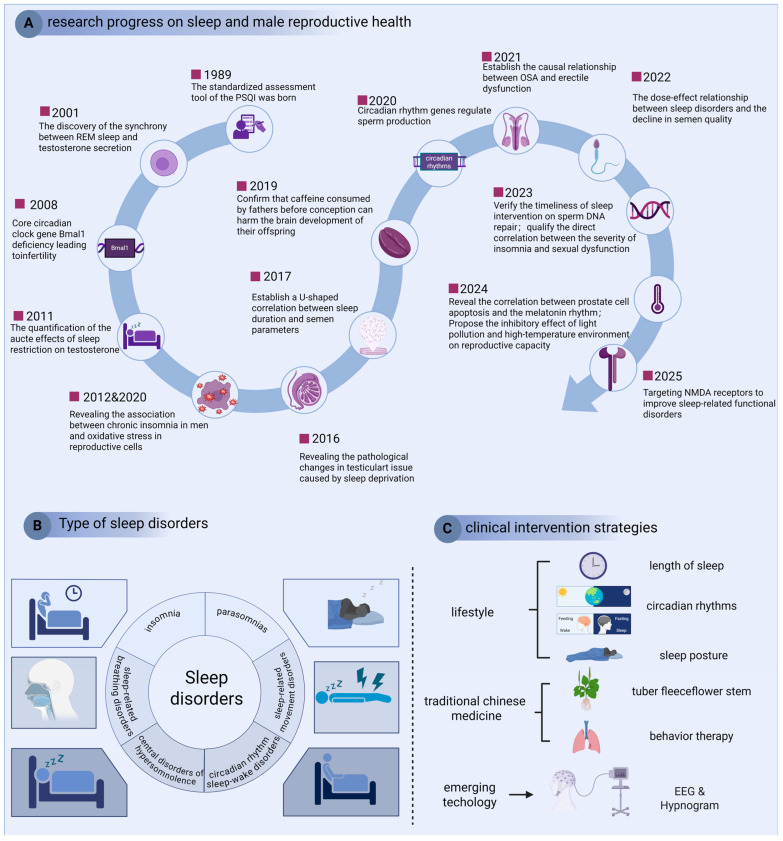
Response of male health to sleep disorders. Research over the past 25 years have indicated melatonin secretion, light rhythm and intensity, adenosine metabolism, and body temperature regulation mediating sleep regulation of male health; the classification of sleep disorders defined by the American Academy of Sleep Medicine (AASM) ranges from insomnia disorder to sleep-related movement disorders; and clinical interventions are mainly based on lifestyle. SQI, Pittsburgh Sleep Quality Index; REM, Rapid eye movement; OSA, Obstructive sleep apnea; NMDA, N-Methyl-D-aspartic acid; EEG, Electroencephalogram.

Emerging evidence underscores the critical association between sleep and male reproductive health. However, contemporary epidemiological data reveal a concerning escalation of sleep disorders in male populations. The American Academy of Sleep Medicine categorizes these disorders into six principal classifications: insomnia, sleep-related breathing disorders, central hypersomnia, circadian rhythm sleep–wake disorders, parasomnias, and sleep-related movement disorders (Figure 4) [96]. Longitudinal analyses demonstrate a progressive decline in sleep duration among American males, with mean nightly sleep decreasing from 7.40 to 7.18 h over the past two decades. Notably, the prevalence of short sleepers (≤6 h) has risen significantly from 22.3% to 29.2% during this period [97]. Although temporal stability was observed between 2004 and 2012, the sustained upward trajectory in sleep deprivation rates presents substantial public health challenges.

Recent studies demonstrate distinct organ-specific consequences of sleep disorders within the urinary system. The kidneys are the most frequently affected organs within the urinary system. Individuals maintaining optimal sleep patterns (score = 5) showed 23% lower CKD incidence compared with those with poor sleep hygiene (score ≤ 1) (HR = 0.77, *p* < 0.001) [98]. Additionally, sleep duration demonstrated a nonlinear U-shaped association with CKD risk. Both short (≤6 h) and prolonged sleep (≥8 h) durations increased CKD risk by 13–45% compared to 7 h sleepers (HR = 1.07–1.45; OR = 1.13–1.25), with maximal vulnerability observed at ≤5 h (OR = 1.42) [99]. Mechanistically, sleep deprivation and insomnia directly impaired glomerular filtration capacity through serum creatinine elevation (short sleep: β = 0.14 mg/dL; insomnia: β = 0.09 mg/dL; both *p* < 0.05) [100]. Intriguingly, compensatory sleep strategies demonstrated significant renal protective effects. Weekend sleep extension (>7 h deficit recovery) reduced CKD risk by 56% (OR = 0.44, *p* = 0.03), while systematic napping attenuated sleep debt consequences (β = −0.21 for eGFR decline, *p* = 0.02) [101]. For nephrolithiasis pathogenesis, sleep disturbances followed a cumulative dose–response pattern. Specific impairments—particularly sleep initiation difficulty (OR = 1.68, 95% CI 1.32–2.14), inadequate duration (HR = 1.13 per hour deficit), and poor sleep quality (HR = 1.19 per SD decrease)—independently elevated calculi risk. Each additional sleep disorder compounded the probability by 14–68% through circadian disruption of urinary citrate excretion (*p* < 0.001) [102,103]. In contrast to renal associations, sleep duration showed no significant association with prostate carcinogenesis (RR = 0.88–0.99; OR = 1.18, *p* = 0.427) [104,105]. However, sleep quality degradation significantly predicted benign prostatic hyperplasia progression (β = 0.34 IPSS points per sleep quality unit decline, *p* < 0.001), potentially mediated through autonomic nervous system dysregulation (Figure 4) [106].

Accumulating evidence establishes quantifiable associations between sleep quality and male reproductive parameters through validated Pittsburgh Sleep Quality Index (PSQI) assessments. Multivariable regression analysis reveals that each 1-point elevation in PSQI total score corresponds to significant reductions in semen quality metrics: 9.287-unit decline in total sperm motility (95% CI −12.05 to −6.52), 9.193-unit decrease in forward motility, and 8-unit reduction in concentration [107]. Notably, these sleep-related impairments exhibit compounded effects through dual pathways: insomnia severity demonstrates a dose-dependent inverse correlation with sexual satisfaction (r = −0.167, *p* < 0.01), while concomitant declines in sperm quality parameters synergistically exacerbate reproductive dysfunction [108]. Sleep duration also manifests a U-shaped association with seminal volume (*p* = 0.002) [109]. Both short (<6 h) and prolonged (>9 h) sleep durations reducing ejaculate volume by 12% (95% CI −22% to −0.68%) and 3.9% (95% CI −7.3% to −0.44%), respectively [109]. Mechanistic investigations implicate oxidative stress pathways in this relationship: chronic insomnia correlates with diminished glutathione peroxidase activity (−38% vs. controls, *p* = 0.007) and elevated lipid peroxidation markers (+52%, *p* = 0.003), creating a pro-oxidant microenvironment detrimental to germ cell viability [110]. Pathological sleep disorders exert more pronounced effects. Each unit increase in obstructive sleep apnea (OSA) severity associates with 0.23 SD decrease in sperm vitality (*p* = 0.018) and 0.19 SD reduction in total motility (*p* = 0.025) [103]. Comparative analysis reveals OSA patients exhibit 22.7% lower progressive motility (30.9% ± 23.2% vs. 53.6% ± 11.1%, *p* < 0.001) alongside impaired sexual function, evidenced by 16.7% decline in IIEF scores (25 vs. 30) and 30% reduction in libido (7 vs. 10) [108]. Circadian regulation emerges as a critical modulator. Genetic ablation studies demonstrate Bmal1-deficient mice develop complete infertility (*p* < 0.001), highlighting core clock genes’ reproductive relevance [111]. Clinical data corroborate these findings: subjects maintaining pre-22:30 bedtimes exhibit 2.75-fold higher odds of normal sperm quality (95% CI 1.1–7.1), whereas post-midnight sleepers show significantly diminished sperm counts (−41%, *p* = 0.012) [112]. Environmental chronodisruptors like light pollution may potentiate these effects through circadian desynchronization [113]. Notably, sleep-sperm DNA integrity relationships follow U-curve dynamics: extreme sleep durations associate with 5% elevation in DNA fragmentation index (95% CI −1 to 13%) and 6% increase in free testosterone (95% CI 0–13%) [114]. Although sleep-onset latency prolongation correlates with 33% semen volume reduction (3.0→2.0 mL, *p* = 0.045), multivariate analysis indicates stronger effects on motility parameters (β = 0.78) versus secretory functions (β = 0.32) [115].

The findings of these studies have provided substantial evidence to support the hypothesis that effective sleep management is a critical component of male reproductive health. However, Sleep deprivation disrupts circadian rhythms, activating the hypothalamic–pituitary–adrenal axis. Elevated glucocorticoids inhibit the hypothalamus’s release of GnRH, thereby suppressing pituitary gonadotropin and testicular testosterone synthesis. Concurrently, reduced melatonin secretion due to sleep disturbances diminishes its antioxidant and protective effects, further exacerbating testicular dysfunction [116]. The maintenance of sleep duration at 7–8 h and the enhancement of sleep quality have emerged as pivotal strategies in the prevention of renal diseases. Concurrently, lifestyle modifications, integrative medicine approaches, and the application of emerging technologies have demonstrated the potential to mitigate the adverse effects of sleep disorders on male reproductive health (Figure 4). However, given the heterogeneity in the sensitivity of different organs to sleep disorders, the development of targeted intervention strategies is imperative.

## 6. Profiles of Adverse Outcomes in Men Under Sedentary: Burden of Genitourinary Diseases and Threat to Life Expectancy

Sedentary behavior [117], operationally defined as wakeful activities characterized by low energy expenditure (≤1.5 metabolic equivalents) in seated or reclined postures, has become a pervasive health challenge in contemporary populations with marked demographic disparities. Sedentary metabolic derangements stem from multisystem pathophysiological interactions: diminished skeletal muscle contractile frequency induces functional impairment of lipoprotein lipase (LPL), leading to a 31% reduction in postprandial triglyceride clearance efficacy (*p* < 0.001) [118]. Prolonged sitting contributes to obesity by reducing energy expenditure and promoting visceral fat accumulation. Obesity subsequently triggers hormonal imbalances, such as decreased testosterone levels and increased, estrogen thereby impairing sperm production and male fertility [119]. The primary manifestations of sedentary behavior on male health encompass the urinary system, reproductive function, and mortality.

**Figure 5:**
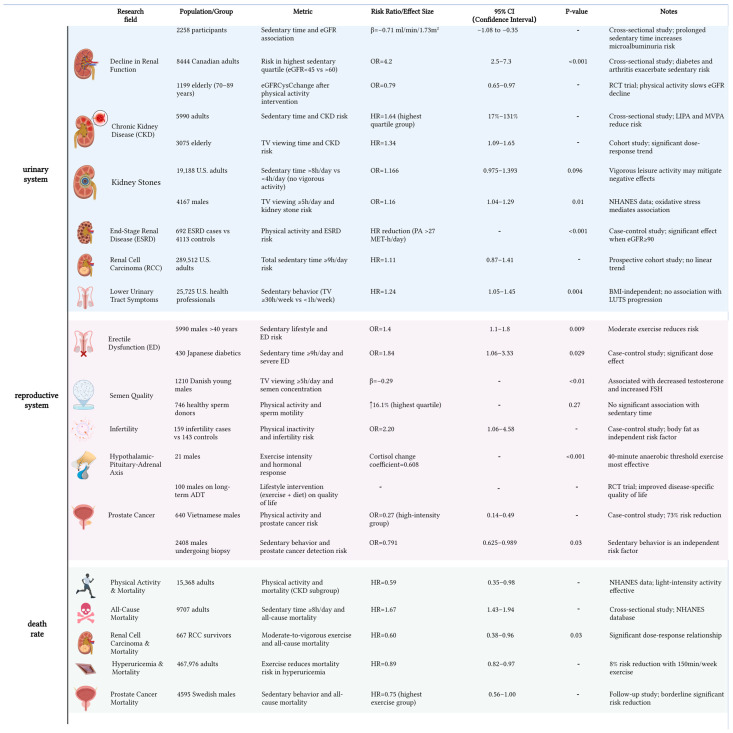
Epidemiological burden of sedentary behavior on male health. Summarized from urinary system, reproductive system and mortality. Effects of sedentary behavior across different races and age-stratified males were investigated based on pooled cohort analyses. The observed adverse outcomes encompass pathologies spanning the entire genitourinary tract and demonstrate elevated mortality risk. These findings were robustly demonstrated to be statistically significant across multiple parameters. CKD, Chronic kidney disease; ESRD, End-stage renal disease; RCC, Renal cell carcinoma; ED, Erectile dysfunction; ADT, Androgen deprivation therapy; RCC, Renal cell carcinoma; eGFR, Estimated glomerular filtration rate; RCT, Randomized controlled trial; LIPA, Lysosomal acid lipase; MVPA, Moderate to vigorous physical activity; NHANES, National Health and Nutrition Examination Survey; BMI, Body mass index; LUTS, Lower urinary tract symptoms; FSH, Follicle-stimulating hormone.

Prolonged sedentary behavior exerts detrimental effects on urinary system homeostasis, with particular pathophysiological implications for kidney (Figure 5). Canadian cohort data stratified by baseline kidney function demonstrate a dose-dependent relationship between sitting time and renal impairment risk [120]. Individuals with advanced renal insufficiency (eGFR < 45 mL/min/1.73 m^2^) exhibit 4.2-fold elevated risk (95% CI 2.5–7.3), while moderate renal dysfunction (eGFR 45–60 mL/min/1.73 m^2^) shows 1.7-fold risk elevation (95% CI 1.2–2.3). This gradient pattern persists across ethnic groups, as evidenced by accelerated renal decline in US Hispanic/Latino populations (−0.06% eGFR reduction per sedentary hour; 95% CI −0.10 to −0.02) [121]. Notably, sex-specific susceptibility patterns emerge from multinational datasets. Female subjects exceeding 8 h daily sitting thresholds demonstrate significantly greater chronic kidney disease progression than males (*p* < 0.01) [122]. Conversely, Korean epidemiological surveys reveal tentative associations between physical inactivity and renal dysfunction in adult males, though lacking statistical significance (*p* > 0.05) [123]. These divergent profiles suggest potential gender dimorphism in sedentary-induced nephropathy mechanisms. When evaluating broader urological outcomes, nonlinear associations become apparent. Comparative analysis reveals a U-shaped relationship between sitting duration and nephrolithiasis risk: 34.1% risk reduction occurs at 6–8 h/day (OR = 0.659, 95% CI 0.457–0.950) versus < 6 h, yet transitions to 12.3% elevation beyond 8 h (OR = 1.123), albeit nonsignificant (*p* = 0.571) [124]. Prostatic inflammation exhibits linear progression, with weekly sitting exceeding 30 h conferring 24% elevated risk (HR = 1.24, 95% CI 1.05–1.45) relative to <1 h controls [125]. Methodological variations in sedentary behavior research—including divergent operational definitions (3–12 h/day thresholds) and measurement tools (self-reports vs. accelerometry)—may explain outcome inconsistencies. Current limitations extend to underpowered cohorts (<10,000 participants in 83% studies) and incomplete confounder adjustment (activity patterns, sociocultural factors), compromising causal attribution. To bridge these gaps, we advocate: standardized metrics incorporating metabolic biomarkers; multinational cohorts (N ≥ 10,000) with sex-stratified designs; and risk-adapted exercise protocols for vulnerable subgroups (e.g., women with incipient renal decline). These priorities aim to decode biological mechanisms and advance precision prevention frameworks.

The association between sedentary behavior and male fertility also remains controversial in current research (Figure 5). While population-level evidence indicates no statistically significant direct correlation between sedentary lifestyle and infertility risk (adjusted OR = 1.20, 95% CI 0.55–2.61, *p* = 0.63), emerging data suggest potential indirect pathways mediated through metabolic alterations. Notably, individuals with elevated adiposity demonstrate a 2.83-fold increased infertility risk (95% CI 1.31–6.10, *p* < 0.01), implying adipose-related metabolic dysregulation may constitute a critical mediating mechanism [126]. Intriguingly, behavior-specific analyses reveal differential impacts: prolonged television viewing (>5 hr/day) associates with 28.8% reduced sperm concentration (37 vs. 52 million/mL) and 34.2% lower total sperm count (104 vs. 158 million), potentially attributable to localized thermal stress or oxidative damage mechanisms [127]. This contrasts with broader epidemiological findings showing no significant correlations between total sedentary duration and conventional semen parameters. Prospective cohort analyses confirm nonsignificant associations for sperm concentration (*p* = 0.37), vitality (*p* = 0.92), and morphology (*p* = 0.16) [128], with subsequent studies replicating these null findings for motility parameters (progressive motility *p* = 0.69; total motility *p* = 0.53) [129]. Notably, a critical interaction emerges between physical activity and sedentarism. Active individuals exhibit superior sperm motility profiles (progressive motility *p* = 0.03; total motility *p* = 0.03) compared to sedentary counterparts, despite comparable sperm concentrations (*p* = 0.37) and total counts (*p* = 0.82) [130]. This dissociation suggests sedentary behavior may preferentially impair functional rather than quantitative aspects of sperm biology, potentially through inactivity-induced metabolic perturbations.

Existing studies demonstrate substantial variations in sedentary-related mortality outcomes across chronic disease subtypes (Figure 5). Our stratified analysis demonstrates differential patterns in CKD populations: while physical activity levels showed no significant inverse correlation with all-cause mortality (*p* > 0.3) [131], prolonged sitting (≥8 h/day) independently elevated mortality risk by 67% (HR = 1.67, 95% CI 1.32–2.11) [132]. Notably, renal function biomarkers (eGFR and urine albumin-to-creatinine ratio [UACR]) emerged as significant predictors in this dose–response relationship. Subgroup analyses revealed distinct protective effects of exercise across clinical populations. Among renal cell carcinoma survivors, high-intensity exercise (≥7 h/week) conferred a 40% mortality reduction (HR = 0.60, 95% CI 0.47–0.76), contrasting with the null association observed for prolonged television viewing [133]. Similarly, patients with hyperuricemia achieving recommended exercise thresholds experienced an 11% lower mortality risk (HR = 0.89, 95% CI 0.82–0.97) [134]. Intriguingly, while sedentary time showed no direct mortality association in prostate cancer patients (*p* = 0.24), sufficient exercisers exhibited an 11% survival advantage over inactive counterparts (ΔHR = 0.89, 95% CI 0.83–0.95), suggesting partial mitigation of sedentarism’s detrimental effects through compensatory physical activity [135].

## 7. Conclusions

Male genitourinary health faces escalating threats from a complex matrix of non-traditional factors, including environmental contaminants and behavioral risks. These agents heighten disease susceptibility through distinct yet interconnected pathways: epigenetic toxicity, DNA fragmentation, hormonal suppression, hyperthermia, and circadian rhythm dysregulation. Interactions between factors range from antagonism to synergistic potentiation, complicating risk prediction. Individual vulnerability varies significantly due to genetic predispositions and heterogeneous environmental exposures across the life course. Critically, certain exposures induce transgenerational harm via epigenetic mechanisms, compromising not only the exposed individual but also offspring phenotypes. This underscores the imperative to address modifiable risks to mitigate reversible damage and intergenerational health crises.

Despite deepening scientific understanding, the actual spectrum of subclinical exposures encountered in real-world settings is vastly broader and more intricate. Addressing this complexity necessitates focused research advances in several critical directions: First, elucidating the mechanisms underlying the combined effects of multi-factorial exposures and their interactions with behavioral co-factors. Second, developing and validating targeted intervention strategies that leverage windows of reversibility to mitigate or repair damage. Third, establishing integrated high precision risk assessment frameworks that synthesize exposomic data, large-scale cohort analyses, and validated individual biomarkers. Fourth, prospectively investigating the long-term and transgenerational health consequences of parental exposures on offspring. Only through such multidimensional and systems-oriented research can evidence-based preventive paradigms and effective clinical interventions be robustly developed to safeguard male genitourinary health.

## Abbreviations

The following abbreviations are used in this manuscript:

5-HT	5-Hydroxytryptamine
AASM	American Academy of Sleep Medicine
ACEI	Angiotensin-Converting Enzyme Inhibitor
ADH	Alcohol Dehydrogenase
ADT	Androgen Deprivation Therapy
ALDH2	Aldehyde Dehydrogenase 2
AMPK	AMP-activated Protein Kinase
AOR	Adjusted Odds Ratio
ATG5	Autophagy-related gene 5
ATG7	Autophagy-related gene 7
BMI	Body Mass Index
BPA	Bisphenol A
CAT	Catalase
CGRP	Calcitonin Gene-Related Peptide
CKD	Chronic Kidney Disease
COX-2	Cyclooxygenase-2
CYP2E1	Cytochrome P450 2E1
DEHP	Di(2-ethylhexyl) Phthalate
DNA	Deoxyribonucleic Acid
ED	Erectile Dysfunction
EDCs	Endocrine-Disrupting Chemicals
EEG	Electroencephalogram
eGFR	Estimated Glomerular Filtration Rate
ESRD	End-stage Renal Disease
FSH	Follicle-Stimulating Hormone
fPSA	Free Prostate-Specific Antigen
GABA	Gamma-Aminobutyric Acid
GnRH	Gonadotropin-Releasing Hormone
HO-1	Heme Oxygenase-1
HR	Hazard Ratio
HSD17B3	Hydroxysteroid 17-beta Dehydrogenase 3
IIEF	International Index of Erectile Function
JAK-STAT	Janus Kinase-Signal Transducer and Activator of Transcription
LH	Luteinizing Hormone
LIPA	Lysosomal Acid Lipase
LPL	Lipoprotein Lipase
LPS	Lipopolysaccharide
LUTS	Lower Urinary Tract Symptoms
MCLR	Microcystin-LR
MPs	Microplastics
MVPA	Moderate to Vigorous Physical Activity
MyD88	Myeloid Differentiation Primary Response 88
NF-κB	Nuclear factor kappa-light-chain-enhancer of activated B cells
NHANES	National Health and Nutrition Examination Survey
NMDA	N-Methyl-D-aspartic acid
NQO1	Nitrosylquinoline Oxidase 1
Nrf1	Nuclear Factor Erythroid 2-Related Factor 1
NPs	Nanoplastics
OAB	Overactive Bladder
OR	Odds Ratio
OSA	Obstructive Sleep Apnea
PCA	Protocatechuic Acid
PE	Polyethylene
PGE2	Prostaglandin E2
PGC-1α	Peroxisome Proliferator-activated receptor Gamma Coactivator 1-alpha
PP	Polypropylene
PRC2	Polycomb Repressive Complex 2
PS	Polystyrene
PSQI	Pittsburgh Sleep Quality Index
PVC	Polyvinyl Chloride
RCC	Renal Cell Carcinoma
RCT	Randomized Controlled Trial
REM	Rapid Eye Movement
RNAPⅡ	RNA Polymerase II
RR	Relative Risk
SCO	Sertoli Cell-Only Syndrome
SHBG	Sex Hormone-Binding Globulin
SMD	Standardized Mean Difference
SOD2	Superoxide Dismutase 2
TLR4	Toll-like receptor 4
TRIF	TIR-domain-containing adapter-inducing interferon-β
UACR	Urine Albumin-to-Creatinine Ratio
UTI	Urinary Tract Infection
SAM	S-Adenosyl methionine
WHO	World Health Organization

## References

[1] White A., Connell R., Griffith D.M., Baker P. (2023). Defining “Men’s Health”. Int. J. Men’s Soc. Community Health.

[2] Agarwal A., Mulgund A., Hamada A., Chyatte M.R. (2015). A unique view on male infertility around the globe. Reprod. Biol. Endocrinol..

[3] Luo X., Yin C., Shi Y., Du C., Pan X. (2023). Global trends in semen quality of young men: A systematic review and regression analysis. J. Assist. Reprod. Genet..

[4] Sung H., Ferlay J., Siegel R.L., Laversanne M., Soerjomataram I., Jemal A., Bray F. (2021). Global Cancer Statistics 2020: GLOBOCAN Estimates of Incidence and Mortality Worldwide for 36 Cancers in 185 Countries. CA Cancer J. Clin..

[5] Schmidt C.W. (2018). Chips off the Old Block: How a Father’s Preconception Exposures Might Affect the Health of His Children. Environ. Health Perspect..

[6] Wang Z., Fang Y., Zhang X. (2024). Impact of Social Capital on Health Behaviors of Middle-Aged and Older Adults in China-An Analysis Based on CHARLS2020 Data. Healthcare.

[7] The Lancet (2019). Icd-11. Lancet.

[8] Tesarik J. (2025). Lifestyle and Environmental Factors Affecting Male Fertility, Individual Predisposition, Prevention, and Intervention. Int. J. Mol. Sci..

[9] The Lancet (2021). Tobacco control: Far from the finish line. Lancet.

[10] Zhang M., Yang L., Wang L., Jiang Y., Huang Z., Zhao Z., Zhang X., Li Y., Liu S., Li C. (2022). Trends in smoking prevalence in urban and rural China, 2007 to 2018: Findings from 5 consecutive nationally representative cross-sectional surveys. PLoS Med..

[11] Shang B., Yao Y., Yin H., Xie Y., Yang S., You X., Liu H., Wang M., Ma J. (2024). In utero, childhood, and adolescence tobacco smoke exposure, physical activity, and chronic kidney disease incidence in adulthood: Evidence from a large prospective cohort study. BMC Med..

[12] Molino A.R., Jerry-Fluker J., Atkinson M.A., Furth S.L., Warady B.A., Ng D.K. (2021). Correction to: The association of alcohol, cigarette, e-cigarette, and marijuana use with disease severity in adolescents and young adults with pediatric chronic kidney disease. Pediatr. Nephrol..

[13] Ito K., Maeda T., Tada K., Takahashi K., Yasuno T., Masutani K., Mukoubara S., Arima H., Nakashima H. (2020). The role of cigarette smoking on new-onset of chronic kidney disease in a Japanese population without prior chronic kidney disease: Iki epidemiological study of atherosclerosis and chronic kidney disease (ISSA-CKD). Clin. Exp. Nephrol..

[14] Matsumoto A., Nagasawa Y., Yamamoto R., Shinzawa M., Yamazaki H., Shojima K., Shinmura K., Isaka Y., Iseki K., Yamagata K. (2024). Cigarette smoking and progression of kidney dysfunction: A longitudinal cohort study. Clin. Exp. Nephrol..

[15] Yang X., Chen H., Zhang S., Chen X., Sheng Y., Pang J. (2023). Association of cigarette smoking habits with the risk of prostate cancer: A systematic review and meta-analysis. BMC Public Health.

[16] Al-Fayez S., El-Metwally A. (2023). Cigarette smoking and prostate cancer: A systematic review and meta-analysis of prospective cohort studies. Tob. Induc. Dis..

[17] Jimenez-Mendoza E., Vazquez-Salas R.A., Barrientos-Gutierrez T., Reynales-Shigematsu L.M., Labra-Salgado I.R., Manzanilla-Garcia H.A., Torres-Sanchez L.E. (2018). Smoking and prostate cancer: A life course analysis. BMC Cancer.

[18] Zhao X., Wang Y., Liang C. (2022). Cigarette smoking and risk of bladder cancer: A dose-response meta-analysis. Int. Urol. Nephrol..

[19] Masaoka H., Matsuo K., Oze I., Kimura T., Tamakoshi A., Sugawara Y., Tsuji I., Sawada N., Tsugane S., Ito H. (2023). Cigarette Smoking, Smoking Cessation, and Bladder Cancer Risk: A Pooled Analysis of 10 Cohort Studies in Japan. J. Epidemiol..

[20] Jee Y., Jung K.J., Back J.H., Lee S.M., Lee S.H. (2020). Trajectory of smoking and early bladder cancer risk among Korean young adult men. Cancer Causes Control.

[21] Sharma R., Biedenharn K.R., Fedor J.M., Agarwal A. (2013). Lifestyle factors and reproductive health: Taking control of your fertility. Reprod. Biol. Endocrinol..

[22] Bao H.Q., Sun L., Yang X.N., Ding J., Ma S.Y., Yang L., Xu X.O. (2019). Impact of cigarette smoking on sperm quality and seminal plasma ROS in preconception males. Zhonghua Nan Ke Xue.

[23] Asare-Anane H., Bannison S.B., Ofori E.K., Ateko R.O., Bawah A.T., Amanquah S.D., Oppong S.Y., Gandau B.B., Ziem J.B. (2016). Tobacco smoking is associated with decreased semen quality. Reprod. Health.

[24] Fan S., Zhang Z., Wang H., Luo L., Xu B. (2024). Associations between tobacco inhalation and semen parameters in men with primary and secondary infertility: A cross-sectional study. Front. Endocrinol..

[25] Kimblad A., Ollvik G., Lindh C.H., Axelsson J. (2022). Decreased sperm counts in Swedish users of oral tobacco. Andrology.

[26] Kulaksiz D., Toprak T., Tokat E., Yilmaz M., Ramazanoglu M.A., Garayev A., Sulukaya M., Degirmentepe R.B., Allahverdiyev E., Gul M. (2022). Sperm concentration and semen volume increase after smoking cessation in infertile men. Int. J. Impot. Res..

[27] Guha P., Bandyopadhyaya G., Polumuri S.K., Chumsri S., Gade P., Kalvakolanu D.V., Ahmed H. (2014). Nicotine promotes apoptosis resistance of breast cancer cells and enrichment of side population cells with cancer stem cell-like properties via a signaling cascade involving galectin-3, alpha9 nicotinic acetylcholine receptor and STAT3. Breast Cancer Res. Treat..

[28] Ranjit S., Midde N.M., Sinha N., Patters B.J., Rahman M.A., Cory T.J., Rao P.S., Kumar S. (2016). Effect of Polyaryl Hydrocarbons on Cytotoxicity in Monocytic Cells: Potential Role of Cytochromes P450 and Oxidative Stress Pathways. PLoS ONE.

[29] Hu R., Yang X., Gong J., Lv J., Yuan X., Shi M., Fu C., Tan B., Fan Z., Chen L. (2024). Patterns of alteration in boar semen quality from 9 to 37 months old and improvement by protocatechuic acid. J. Anim. Sci. Biotechnol..

[30] Peacock A., Leung J., Larney S., Colledge S., Hickman M., Rehm J., Giovino G.A., West R., Hall W., Griffiths P. (2018). Global statistics on alcohol, tobacco and illicit drug use: 2017 status report. Addiction.

[31] Finelli R., Mottola F., Agarwal A. (2021). Impact of Alcohol Consumption on Male Fertility Potential: A Narrative Review. Int. J. Environ. Res. Public Health.

[32] Yuan H.C., Yu Q.T., Bai H., Xu H.Z., Gu P., Chen L.Y. (2021). Alcohol intake and the risk of chronic kidney disease: Results from a systematic review and dose-response meta-analysis. Eur. J. Clin. Nutr..

[33] Yang S., Tan W., Wei B., Gu C., Li S., Wang S. (2023). Association between alcohol and urolithiasis: A mendelian randomization study. Urolithiasis.

[34] Vartolomei M.D., Iwata T., Roth B., Kimura S., Mathieu R., Ferro M., Shariat S.F., Seitz C. (2019). Impact of alcohol consumption on the risk of developing bladder cancer: A systematic review and meta-analysis. World J. Urol..

[35] Zhang Y., Qin W. (2024). Relationship between alcohol use and overactive bladder disease: A cross-sectional study of the NHANES 2005–2016. Front. Public Health.

[36] Hong S., Khil H., Lee D.H., Keum N., Giovannucci E.L. (2020). Alcohol Consumption and the Risk of Prostate Cancer: A Dose-Response Meta-Analysis. Nutrients.

[37] Michael J., Howard L.E., Markt S.C., De Hoedt A., Bailey C., Mucci L.A., Freedland S.J., Allott E.H. (2018). Early-Life Alcohol Intake and High-Grade Prostate Cancer: Results from an Equal-Access, Racially Diverse Biopsy Cohort. Cancer Prev. Res..

[38] Rao M., Zuo L.D., Fang F., Martin K., Zheng Y., Zhang H.P., Li H.G., Zhu C.H., Xiong C.L., Guan H.T. (2015). Association of Alcohol Consumption with Markers of Prostate Health and Reproductive Hormone Profiles: A Multi-Center Study of 4,535 Men in China. PLoS ONE.

[39] Weng X., Tan W., Wei B., Yang S., Gu C., Wang S. (2023). Interaction between drinking and dietary inflammatory index affects prostate specific antigen: A cross-sectional study. BMC Geriatr..

[40] Gianfrilli D., Ferlin A., Isidori A.M., Garolla A., Maggi M., Pivonello R., Santi D., Sansone A., Balercia G., Granata A.R.M. (2019). Risk behaviours and alcohol in adolescence are negatively associated with testicular volume: Results from the Amico-Andrologo survey. Andrology.

[41] Nguyen-Thanh T., Hoang-Thi A.P., Anh Thu D.T. (2023). Investigating the association between alcohol intake and male reproductive function: A current meta-analysis. Heliyon.

[42] Ricci E., Noli S., Ferrari S., La Vecchia I., Cipriani S., De Cosmi V., Somigliana E., Parazzini F. (2018). Alcohol intake and semen variables: Cross-sectional analysis of a prospective cohort study of men referring to an Italian Fertility Clinic. Andrology.

[43] Bai S., Wan Y., Zong L., Li W., Xu X., Zhao Y., Hu X., Zuo Y., Xu B., Tong X. (2020). Association of Alcohol Intake and Semen Parameters in Men With Primary and Secondary Infertility: A Cross-Sectional Study. Front. Physiol..

[44] Boeri L., Capogrosso P., Ventimiglia E., Pederzoli F., Cazzaniga W., Chierigo F., Deho F., Montanari E., Montorsi F., Salonia A. (2019). Heavy cigarette smoking and alcohol consumption are associated with impaired sperm parameters in primary infertile men. Asian J. Androl..

[45] Greenberg D.R., Bhambhvani H.P., Basran S.S., Salazar B.P., Rios L.C., Li S.J., Chen C.H., Mochly-Rosen D., Eisenberg M.L. (2022). ALDH2 Expression, Alcohol Intake and Semen Parameters among East Asian Men. J. Urol..

[46] Mai H., Ke J., Zheng Z., Luo J., Li M., Qu Y., Jiang F., Cai S., Zuo L. (2023). Association of diet and lifestyle factors with semen quality in male partners of Chinese couples preparing for pregnancy. Reprod. Health.

[47] Karmon A.E., Toth T.L., Chiu Y.H., Gaskins A.J., Tanrikut C., Wright D.L., Hauser R., Chavarro J.E., Earth Study T. (2017). Male caffeine and alcohol intake in relation to semen parameters and in vitro fertilization outcomes among fertility patients. Andrology.

[48] Sanchez M.C., Fontana V.A., Galotto C., Cambiasso M.Y., Sobarzo C.M.A., Calvo L., Calvo J.C., Cebral E. (2018). Murine sperm capacitation, oocyte penetration and decondensation following moderate alcohol intake. Reproduction.

[49] Borges E., Braga D., Provenza R.R., Figueira R.C.S., Iaconelli A., Setti A.S. (2018). Paternal lifestyle factors in relation to semen quality and in vitro reproductive outcomes. Andrologia.

[50] Ricci E., Al Beitawi S., Cipriani S., Candiani M., Chiaffarino F., Vigano P., Noli S., Parazzini F. (2017). Semen quality and alcohol intake: A systematic review and meta-analysis. Reprod. Biomed. Online.

[51] Shi X., Chan C.P.S., Waters T., Chi L., Chan D.Y.L., Li T.C. (2018). Lifestyle and demographic factors associated with human semen quality and sperm function. Syst. Biol. Reprod. Med..

[52] Hu R., Yang X., Wang L., Su D., He Z., Li J., Gong J., Zhang W., Ma S., Shi M. (2024). Gut microbiota dysbiosis and oxidative damage in high-fat diet-induced impairment of spermatogenesis: Role of protocatechuic acid intervention. Food Front..

[53] Duca Y., Aversa A., Condorelli R.A., Calogero A.E., La Vignera S. (2019). Substance Abuse and Male Hypogonadism. J. Clin. Med..

[54] Aliabad M.K., Nassiri M., Kor K. (2019). Microplastics in the surface seawaters of Chabahar Bay, Gulf of Oman (Makran Coasts). Mar. Pollut. Bull..

[55] Batel A., Linti F., Scherer M., Erdinger L., Braunbeck T. (2016). Transfer of benzo[a]pyrene from microplastics to Artemia nauplii and further to zebrafish via a trophic food web experiment: CYP1A induction and visual tracking of persistent organic pollutants. Environ. Toxicol. Chem..

[56] Hu L., Zhao Y., Xu H. (2022). Trojan horse in the intestine: A review on the biotoxicity of microplastics combined environmental contaminants. J. Hazard. Mater..

[57] Cortes-Arriagada D., Ortega D.E., Miranda-Rojas S. (2023). Mechanistic insights into the adsorption of endocrine disruptors onto polystyrene microplastics in water. Environ. Pollut..

[58] Ragusa A., Svelato A., Santacroce C., Catalano P., Notarstefano V., Carnevali O., Papa F., Rongioletti M.C.A., Baiocco F., Draghi S. (2021). Plasticenta: First evidence of microplastics in human placenta. Environ. Int..

[59] Ijaz M.U., Rafi Z., Hamza A., Sayed A.A., Albadrani G.M., Al-Ghadi M.Q., Abdel-Daim M.M. (2024). Mitigative potential of kaempferide against polyethylene microplastics induced testicular damage by activating Nrf-2/Keap-1 pathway. Ecotoxicol. Environ. Saf..

[60] Kwak J.I., An Y.J. (2021). Microplastic digestion generates fragmented nanoplastics in soils and damages earthworm spermatogenesis and coelomocyte viability. J. Hazard. Mater..

[61] Li T., Bian B., Ji R., Zhu X., Wo X., Song Q., Li Z., Wang F., Jia Y. (2024). Polyethylene Terephthalate Microplastic Exposure Induced Reproductive Toxicity Through Oxidative Stress and p38 Signaling Pathway Activation in Male Mice. Toxics.

[62] Yang Y.K., Ge S.J., Su Q.L., Chen J.J., Wu J., Kang K. (2024). Effects of Polyvinyl Chloride Microplastics on the Reproductive System, Intestinal Structure, and Microflora in Male and Female Mice. Vet. Sci..

[63] Zheng Y., Nowack B. (2022). Meta-analysis of Bioaccumulation Data for Nondissolvable Engineered Nanomaterials in Freshwater Aquatic Organisms. Environ. Toxicol. Chem..

[64] Hu M., Palic D. (2020). Micro- and nano-plastics activation of oxidative and inflammatory adverse outcome pathways. Redox Biol..

[65] Ji Z., Huang Y., Feng Y., Johansen A., Xue J., Tremblay L.A., Li Z. (2021). Effects of pristine microplastics and nanoplastics on soil invertebrates: A systematic review and meta-analysis of available data. Sci. Total Environ..

[66] Jin H., Yan M., Pan C., Liu Z., Sha X., Jiang C., Li L., Pan M., Li D., Han X. (2022). Chronic exposure to polystyrene microplastics induced male reproductive toxicity and decreased testosterone levels via the LH-mediated LHR/cAMP/PKA/StAR pathway. Part. Fibre Toxicol..

[67] Lee S., Kang K.K., Sung S.E., Choi J.H., Sung M., Seong K.Y., Lee S., Yang S.Y., Seo M.S., Kim K. (2022). Toxicity Study and Quantitative Evaluation of Polyethylene Microplastics in ICR Mice. Polymers.

[68] Jin H., Ma T., Sha X., Liu Z., Zhou Y., Meng X., Chen Y., Han X., Ding J. (2021). Polystyrene microplastics induced male reproductive toxicity in mice. J. Hazard. Mater..

[69] Park E.J., Han J.S., Park E.J., Seong E., Lee G.H., Kim D.W., Son H.Y., Han H.Y., Lee B.S. (2020). Repeated-oral dose toxicity of polyethylene microplastics and the possible implications on reproduction and development of the next generation. Toxicol. Lett..

[70] Ijaz M.U., Shahzadi S., Samad A., Ehsan N., Ahmed H., Tahir A., Rehman H., Anwar H. (2021). Dose-Dependent Effect of Polystyrene Microplastics on the Testicular Tissues of the Male Sprague Dawley Rats. Dose Response.

[71] Fu G., Wu Q., Dai J., Lu S., Zhou T., Yang Z., Shi Y. (2024). piRNA array analysis provide insight into the mechanism of DEHP-induced testicular toxicology in pubertal male rats. Ecotoxicol. Environ. Saf..

[72] Han B., Hua L., Yu S., Ge W., Huang C., Tian Y., Li C., Yan J., Qiao T., Guo J. (2025). Revealing the core suppression effects of various Di (2-ethylhexyl) phthalate exposure on early meiosis progression in postnatal male mice via single-cell RNA sequencing. Ecotoxicol. Environ. Saf..

[73] Kitaoka M., Hirai S., Terayama H., Naito M., Qu N., Hatayama N., Miyaso H., Matsuno Y., Komiyama M., Itoh M. (2013). Effects on the local immunity in the testis by exposure to di-(2-ethylhexyl) phthalate (DEHP) in mice. J. Reprod. Dev..

[74] Bolling A.K., Ovrevik J., Samuelsen J.T., Holme J.A., Rakkestad K.E., Mathisen G.H., Paulsen R.E., Korsnes M.S., Becher R. (2012). Mono-2-ethylhexylphthalate (MEHP) induces TNF-alpha release and macrophage differentiation through different signalling pathways in RAW264.7 cells. Toxicol. Lett..

[75] Fang Z., Jin Z., Zhao Q., Weng J., Zhang Z., Yang Y., Jiang H. (2025). Multi-omics revealed activation of TNF-alpha induced apoptosis signaling pathway in testis of DEHP treated prepubertal male rat. Reprod. Toxicol..

[76] Meng Y., Lin R., Wu F., Sun Q., Jia L. (2018). Decreased Capacity for Sperm Production Induced by Perinatal Bisphenol A Exposure Is Associated with an Increased Inflammatory Response in the Offspring of C57BL/6 Male Mice. Int. J. Environ. Res. Public Health.

[77] Shi M., Lin Z., Ye L., Chen X., Zhang W., Zhang Z., Luo F., Liu Y., Shi M. (2020). Estrogen receptor-regulated SOCS3 modulation via JAK2/STAT3 pathway is involved in BPF-induced M1 polarization of macrophages. Toxicology.

[78] Gao Z., Liu S., Tan L., Gao X., Fan W., Ding C., Li M., Tang Z., Shi X., Luo Y. (2022). Testicular toxicity of bisphenol compounds: Homeostasis disruption of cholesterol/testosterone via PPARalpha activation. Sci. Total Environ..

[79] Chen X., Dong Y., Tian E., Xie L., Wang G., Li X., Chen X., Chen Y., Lv Y., Ni C. (2018). 4-Bromodiphenyl ether delays pubertal Leydig cell development in rats. Chemosphere.

[80] Wu D., Huang C.J., Jiao X.F., Ding Z.M., Zhang S.X., Miao Y.L., Huo L.J. (2019). Bisphenol AF compromises blood-testis barrier integrity and sperm quality in mice. Chemosphere.

[81] Hassine M.B.H., Venditti M., Rhouma M.B., Minucci S., Messaoudi I. (2023). Combined effect of polystyrene microplastics and cadmium on rat blood-testis barrier integrity and sperm quality. Environ. Sci. Pollut. Res. Int..

[82] Rao G., Qiao B., Zhong G., Li T., Su Q., Wu S., Tang Z., Hu L. (2024). Arsenic and polystyrene-nano plastics co-exposure induced testicular toxicity: Triggers oxidative stress and promotes apoptosis and inflammation in mice. Environ. Toxicol..

[83] Deng Y., Yan Z., Shen R., Huang Y., Ren H., Zhang Y. (2021). Enhanced reproductive toxicities induced by phthalates contaminated microplastics in male mice (*Mus musculus*). J. Hazard. Mater..

[84] Li D., Sun W., Jiang X., Yu Z., Xia Y., Cheng S., Mao L., Luo S., Tang S., Xu S. (2022). Polystyrene nanoparticles enhance the adverse effects of di-(2-ethylhexyl) phthalate on male reproductive system in mice. Ecotoxicol. Environ. Saf..

[85] Ma T., Liu X., Xiong T., Li H., Zhou Y., Liang J. (2023). Polystyrene nanoplastics aggravated dibutyl phthalate-induced blood-testis barrier dysfunction via suppressing autophagy in male mice. Ecotoxicol. Environ. Saf..

[86] Wu H., Liu Q., Yang N., Xu S. (2023). Polystyrene-microplastics and DEHP co-exposure induced DNA damage, cell cycle arrest and necroptosis of ovarian granulosa cells in mice by promoting ROS production. Sci. Total Environ..

[87] Li Y., Liu Y., Chen Y., Yao C., Yu S., Qu J., Chen G., Wei H. (2024). Combined effects of polystyrene nanoplastics and lipopolysaccharide on testosterone biosynthesis and inflammation in mouse testis. Ecotoxicol. Environ. Saf..

[88] Xie X., Deng T., Duan J., Xie J., Yuan J., Chen M. (2020). Exposure to polystyrene microplastics causes reproductive toxicity through oxidative stress and activation of the p38 MAPK signaling pathway. Ecotoxicol. Environ. Saf..

[89] Li S., Wang Q., Yu H., Yang L., Sun Y., Xu N., Wang N., Lei Z., Hou J., Jin Y. (2021). Polystyrene microplastics induce blood-testis barrier disruption regulated by the MAPK-Nrf2 signaling pathway in rats. Environ. Sci. Pollut. Res. Int..

[90] Sussarellu R., Suquet M., Thomas Y., Lambert C., Fabioux C., Pernet M.E., Le Goic N., Quillien V., Mingant C., Epelboin Y. (2016). Oyster reproduction is affected by exposure to polystyrene microplastics. Proc. Natl. Acad. Sci. USA.

[91] Qiang L., Cheng J. (2021). Exposure to polystyrene microplastics impairs gonads of zebrafish (*Danio rerio*). Chemosphere.

[92] Lin Z., Li Z., Ji S., Lo H.S., Billah B., Sharmin A., Han X., Lui W.Y., Tse W.K.F., Fang J.K. (2024). Size-dependent deleterious effects of nano- and microplastics on sperm motility. Toxicology.

[93] Wen Y., Cai J., Zhang H., Li Y., Yu M., Liu J., Han F. (2024). The Potential Mechanisms Involved in the Disruption of Spermatogenesis in Mice byNanoplastics and Microplastics. Biomedicines.

[94] Fang Q., Wang C., Xiong Y. (2024). Polystyrene microplastics induce male reproductive toxicity in mice by activating spermatogonium mitochondrial oxidative stress and apoptosis. Chem. Biol. Interact..

[95] Wu D., Zhang M., Bao T.T., Lan H. (2023). Long-term exposure to polystyrene microplastics triggers premature testicular aging. Part Fibre Toxicol..

[96] Mutlu A., Sisman A.B., Gunaydin S., Balci B.P. (2025). Sleep Disorders in Patients with Epilepsy. Noro Psikiyatr Ars..

[97] Ford E.S., Cunningham T.J., Croft J.B. (2015). Trends in Self-Reported Sleep Duration among US Adults from 1985 to 2012. Sleep.

[98] Geng T., Li X., Ma H., Heianza Y., Qi L. (2022). Adherence to a Healthy Sleep Pattern and Risk of Chronic Kidney Disease: The UK Biobank Study. Mayo Clin. Proc..

[99] Bo Y., Yeoh E.K., Guo C., Zhang Z., Tam T., Chan T.C., Chang L.Y., Lao X.Q. (2019). Sleep and the Risk of Chronic Kidney Disease: A Cohort Study. J. Clin. Sleep. Med..

[100] Zhang Y., Zhong Z., Tang Z., Wang R., Wu J., Na N., Zhang J. (2024). Insomnia and sleep duration for kidney function: Mendelian randomization study. Ren. Fail..

[101] Jiang B., Tang D., Dai N., Huang C., Liu Y., Wang C., Peng J., Qin G., Yu Y., Chen J. (2023). Association of Self-Reported Nighttime Sleep Duration with Chronic Kidney Disease: China Health and Retirement Longitudinal Study. Am. J. Nephrol..

[102] Yan B., Yu J., Fang Q., Qiu H., Shen C., Wang J., Li J., Huang Y., Dai L., Zhi Y. (2024). Association between kidney stones and poor sleep factors in U.S. adults. Medicine.

[103] Wang H., Zhang Y.Q., Yu C.Q., Guo Y., Pei P., Chen J.S., Chen Z.M., Lyu J., Li L. (2022). Associations between sleep status and risk for kidney stones in Chinese adults: A prospective cohort study. Zhonghua Liu Xing Bing Xue Za Zhi.

[104] Li J., Huang Z., Hou J., Sawyer A.M., Wu Z., Cai J., Curhan G., Wu S., Gao X. (2017). Sleep and CKD in Chinese Adults: A Cross-Sectional Study. Clin. J. Am. Soc. Nephrol..

[105] Lozano-Lorca M., Olmedo-Requena R., Vega-Galindo M.V., Vazquez-Alonso F., Jimenez-Pacheco A., Salcedo-Bellido I., Sanchez M.J., Jimenez-Moleon J.J. (2020). Night Shift Work, Chronotype, Sleep Duration, and Prostate Cancer Risk: CAPLIFE Study. Int. J. Environ. Res. Public Health.

[106] Ma K., Dong Q. (2023). Association between sleep quality and benign prostate hyperplasia among middle-aged and older men in India. BMC Public Health.

[107] Du C.Q., Yang Y.Y., Chen J., Feng L., Lin W.Q. (2020). Association Between Sleep Quality and Semen Parameters and Reproductive Hormones: A Cross-Sectional Study in Zhejiang, China. Nat. Sci. Sleep.

[108] Kyrkou K., Alevrakis E., Baou K., Alchanatis M., Poulopoulou C., Kanopoulos C., Vagiakis E., Dikeos D. (2022). Impaired Human Sexual and Erectile Function Affecting Semen Quality, in Obstructive Sleep Apnea: A Pilot Study. J. Pers. Med..

[109] Chen Q., Yang H., Zhou N., Sun L., Bao H., Tan L., Chen H., Ling X., Zhang G., Huang L. (2016). Inverse U-shaped Association between Sleep Duration and Semen Quality: Longitudinal Observational Study (MARHCS) in Chongqing, China. Sleep.

[110] Gulec M., Ozkol H., Selvi Y., Tuluce Y., Aydin A., Besiroglu L., Ozdemir P.G. (2012). Oxidative stress in patients with primary insomnia. Prog. Neuropsychopharmacol. Biol. Psychiatry.

[111] Alvarez J.D., Hansen A., Ord T., Bebas P., Chappell P.E., Giebultowicz J.M., Williams C., Moss S., Sehgal A. (2008). The circadian clock protein BMAL1 is necessary for fertility and proper testosterone production in mice. J. Biol. Rhythm..

[112] Hvidt J.E.M., Knudsen U.B., Zachariae R., Ingerslev H.J., Philipsen M.T., Frederiksen Y. (2020). Associations of bedtime, sleep duration, and sleep quality with semen quality in males seeking fertility treatment: A preliminary study. Basic. Clin. Androl..

[113] Liu P.Y. (2024). Light pollution: Time to consider testicular effects. Front. Toxicol..

[114] Gaml-Sorensen A., Frolich M.K., Brix N., Ernst A., Bonde J.P.E., Hougaard K.S., Tottenborg S.S., Clemmensen P.J., Toft G., Ramlau-Hansen C.H. (2024). Sleep duration and biomarkers of fecundity in young men: A cross-sectional study from a population-based cohort. Andrology.

[115] Vigano P., Chiaffarino F., Bonzi V., Salonia A., Ricci E., Papaleo E., Mauri P.A., Parazzini F. (2017). Sleep disturbances and semen quality in an Italian cross sectional study. Basic. Clin. Androl..

[116] Zhang W., Shi X., Zhang Y., Liu G., Wu X., Huang H., Jiang H., Zhang X. (2023). Attenuation Effect of Recovery Sleep for Impaired Reproductive Function in Male Rats by Sleep Deprivation. World J. Men’s Health.

[117] Wilmot E.G., Edwardson C.L., Achana F.A., Davies M.J., Gorely T., Gray L.J., Khunti K., Yates T., Biddle S.J. Sedentary time in adults and the association with diabetes, cardiovascular disease and death: Systematic review and meta-analysis.

[118] Dunstan D.W., Howard B., Healy G.N., Owen N. (2012). Too much sitting—A health hazard. Diabetes Res. Clin. Pract..

[119] Heydari H., Ghiasi R., Ghaderpour S., Keyhanmanesh R. (2021). The Mechanisms Involved in Obesity-Induced Male Infertility. Curr. Diabetes Rev..

[120] Glavinovic T., Ferguson T., Komenda P., Rigatto C., Duhamel T.A., Tangri N., Bohm C. (2018). CKD and Sedentary Time: Results from the Canadian Health Measures Survey. Am. J. Kidney Dis..

[121] Hannan M., Ricardo A.C., Cai J., Franceschini N., Kaplan R., Marquez D.X., Rosas S.E., Schneiderman N., Sotres-Alvarez D., Talavera G.A. (2021). Sedentary Behavior and Change in Kidney Function: The Hispanic Community Health Study/Study of Latinos (HCHS/SOL). Kidney360.

[122] Bharakhada N., Yates T., Davies M.J., Wilmot E.G., Edwardson C., Henson J., Webb D., Khunti K. (2012). Association of sitting time and physical activity with CKD: A cross-sectional study in family practices. Am. J. Kidney Dis..

[123] Jang Y.S., Park Y.S., Kim H., Hurh K., Park E.C., Jang S.Y. (2023). Association between sedentary behavior and chronic kidney disease in Korean adults. BMC Public Health.

[124] Li Y., Di X., Liu M., Wei J., Li T., Liao B. (2024). Association between daily sitting time and kidney stones based on the National Health and Nutrition Examination Survey (NHANES) 2007–2016: A cross-sectional study. Int. J. Surg..

[125] Mondul A.M., Giovannucci E., Platz E.A. (2020). A Prospective Study of Physical Activity, Sedentary Behavior, and Incidence and Progression of Lower Urinary Tract Symptoms. J. Gen. Intern. Med..

[126] Foucaut A.M., Faure C., Julia C., Czernichow S., Levy R., Dupont C., ALIFERT collaborative group (2019). Sedentary behavior, physical inactivity and body composition in relation to idiopathic infertility among men and women. PLoS ONE.

[127] Priskorn L., Jensen T.K., Bang A.K., Nordkap L., Joensen U.N., Lassen T.H., Olesen I.A., Swan S.H., Skakkebaek N.E., Jorgensen N. (2016). Is Sedentary Lifestyle Associated with Testicular Function? A Cross-Sectional Study of 1,210 Men. Am. J. Epidemiol..

[128] Gaskins A.J., Afeiche M.C., Hauser R., Williams P.L., Gillman M.W., Tanrikut C., Petrozza J.C., Chavarro J.E. (2014). Paternal physical and sedentary activities in relation to semen quality and reproductive outcomes among couples from a fertility center. Hum. Reprod..

[129] Sun B., Messerlian C., Sun Z.H., Duan P., Chen H.G., Chen Y.J., Wang P., Wang L., Meng T.Q., Wang Q. (2019). Physical activity and sedentary time in relation to semen quality in healthy men screened as potential sperm donors. Hum. Reprod..

[130] Lalinde-Acevedo P.C., Mayorga-Torres B.J.M., Agarwal A., du Plessis S.S., Ahmad G., Cadavid A.P., Cardona Maya W.D. (2017). Physically Active Men Show Better Semen Parameters than Their Sedentary Counterparts. Int. J. Fertil. Steril..

[131] Beddhu S., Baird B.C., Zitterkoph J., Neilson J., Greene T. (2009). Physical activity and mortality in chronic kidney disease (NHANES III). Clin. J. Am. Soc. Nephrol..

[132] Wang L., Wu X., Guo Z., Dong Y., Yu B. (2025). Prolonged sitting time and all-cause mortality: The mediating and predictive role of kidney function markers. Ren. Fail..

[133] Schmid D., Matthews C.E., Leitzmann M.F. (2018). Physical activity and sedentary behavior in relation to mortality among renal cell cancer survivors. PLoS ONE.

[134] Chen J.H., Wen C.P., Wu S.B., Lan J.L., Tsai M.K., Tai Y.P., Lee J.H., Hsu C.C., Tsao C.K., Wai J.P. (2015). Attenuating the mortality risk of high serum uric acid: The role of physical activity underused. Ann. Rheum. Dis..

[135] Bonn S.E., Holmberg E., Hugosson J., Balter K. (2020). Is leisure time sitting associated with mortality rates among men diagnosed with localized prostate cancer?. Eur. J. Cancer Prev..

